# Lower Extremity Muscle Activation during the Star Excursion Balance Test in Patients with Chronic Ankle Instability and Copers

**DOI:** 10.3390/medicina59061040

**Published:** 2023-05-28

**Authors:** Yong Ung Kwon

**Affiliations:** Department of Sports Science, Chung-Ang University, Anseong 17546, Republic of Korea; mugika2@gmail.com

**Keywords:** postural control, electromyography, ankle sprain, mean amplitude, kinematics

## Abstract

*Background and Objectives*: The aim of this study was to assess the impact of ankle muscles on performance of the Star Excursion Balance Test (SEBT) among individuals with stable ankles, a history of ankle sprain, and chronic ankle instability (CAI). *Materials and Methods*: Sixty subjects (twenty per group) performed the SEBT in each of the anterior (A), posteromedial (PM), and posterolateral (PL) directions. Normalized maximum reach distance (NMRD) and normalized mean amplitude of the tibialis anterior (NMA_TA), fibularis longus (NMA_FL), and medial gastrocnemius (NMA_MG) were measured during performance of the SEBT. *Results*: Copers have greater NMRD than subjects with stable ankles and those with CAI, and subjects with stable ankles also have greater NMRD than those with CAI in only the PL direction. Subjects with stable ankles and those with CAI showed greater NMA_TA than copers. The A direction showed greater NMA_TA than the PM and PL directions. Copers showed greater NMA_FL than subjects with stable ankles. Subjects with CAI showed greater NMA_MG than copers and subjects with stable ankles. The A and PL directions showed greater NMA_MG than the PM direction. *Conclusions*: Overall, copers and/or subjects with CAI demonstrated altered neuromuscular function by compensating for their ankle muscles when compared to subjects with stable ankles due to a history of ankle sprain.

## 1. Introduction

Ankle sprains are very common injuries that often occur during physical activities [1]. Lateral ankle sprains frequently cause damage to the collateral ligamentous structures of the ankle [2,3]. The anterior talofibular ligament is the weakest and the most commonly injured ligament leading to laxity in the joint, causing functional and mechanical pathologies [2,3]. Functional and mechanical deficits are symptoms of chronic ankle instability (CAI) [4]. As the epidemiology of CAI is unclear, some individuals (copers) do not have recurrent ankle sprains and symptoms of CAI [5]. Understanding the differences among CAI, coper, and control groups is important because researchers believe that copers may have a better coping mechanism for neuromuscular function after a lateral ankle sprain [6,7].

Muscle activation during postural control tests in individuals with CAI has been measured using EMG [8,9,10]. Muscle strength deficits in the evertors, dorsiflexors, and invertors as well as the postural control deficits stated above are present in individuals with CAI [9,11,12]. Moreover, activation of the tibialis anterior (TA), soleus, and fibularis muscles during dynamic movements is higher in individuals with CAI than those with stable ankles [8,9,10]. This can be attributed to the fact that the ankle joint has more laxity and weaker muscle strength after an ankle sprain, so the muscles surrounding the ankle joint must work harder and more rapidly to stabilize the joint to prevent the recurrence of a sprain compared to individuals with stable ankles [2,3,12]. According to these previous studies, muscle activation could be a contributing factor to performance during the Star Excursion Balance Test (SEBT) [9,10,11,12,13,14]. However, the measurement of ankle muscle activities during the SEBT among CAI, coper, and control groups still needs to be added to the literature. The SEBT is a dynamic postural control test that can be accurately used to discriminate neuromuscular function in individuals with CAI and copers by assessing maximal reach distances [15,16,17,18]. The anterior (A), posterolateral (PL), and posteromedial (PM) directions have been the most sensitive directions to detect deficits between individuals with and without CAI [19].

There has been only one study that utilizes EMG and the SEBT to compare copers to individuals with stable ankles and those with CAI [10]. The TA and fibularis longus (FL) muscles presented increased activation in individuals with CAI and copers during the PM reach in the SEBT compared to individuals with stable ankles [10]. However, this study only utilized one of the three reliable directions of the SEBT, eliciting them to use EMG on the TA and FL muscles only [10,19]. Since the gastrocnemius is the primary plantarflexor muscle which may be more related to posterior movement than the TA, it is important to measure it in the PL and PM directions. However, to the best of our knowledge, no study has investigated EMG muscle activations of the TA, FL, and medial gastrocnemius (MG) during the SEBT consisting of the A, PL, and PM directions in copers.

As stated above, the A, PM, and PL directions show high reliability, and the FL, TA, and soleus muscles collectively show high activation in individuals with CAI [8,9,10,19]. However, very few studies investigated the relationship between individuals with CAI and copers during the SEBT [10,20]. Using the three directions in the SEBT and the three muscles in EMG, as stated above, is fundamental to investigate how these muscles contribute to performance of the SEBT in copers compared to individuals with stable ankles and those with CAI. Therefore, the two purposes of this study were to (1) assess the impact of the three main ankle muscles on the maximum reach distance of the SEBT and (2) to investigate differences in performance of the SEBT along with the three directions among the CAI, coper, and control groups. Using EMG to measure activation of the MG, TA, and FL muscles will help to clarify the deficits in the reach distances among the three populations. We hypothesize that muscle activation would be greater in individuals with CAI and copers than in individuals with stable ankles because of the increased need for muscle activation to maximally reach from the pre-existing conditions of muscle weakness and poor balance from CAI and copers. In addition, we expect muscle activation to correlate with reach directions, demonstrating that the A direction primarily activates the TA, while the FL and MG activate more in the PM and PL directions.

## 2. Materials and Methods

### 2.1. Subjects

A total of 60 subjects were recruited for this study: 20 subjects with unilateral CAI (169(9) cm, 73(11) kg, 22(2) yr, CAIT: 19.2(1.3), FAAM ADL: 85.2(1.0)), 20 subjects determined as copers (170(9) cm, 72(15) kg, 23(2) yr, CAIT: 27.9(2.8), FAAM ADL: 98.7(0.8)), and 20 subjects with stable ankles (168(8) cm, 66(12) kg, 22(3) yr, CAIT: 28.8(2.1), FAAM ADL: 99.2(0.7)). All groups were gender-matched groups. To be included in the CAI group, subjects were required to meet the following criteria: (1) a history of at least one significant ankle sprain which involved pain or swelling and at least one interrupted day of desired physical activity, (2) an initial ankle sprain which must have occurred at least one year prior to participation in this study, (3) multiple episodes (≥2) of the ankle giving way within the past six months, and/or (4) feeling of instability, and (5) no history of previous surgeries of musculoskeletal structures or of fracture in the lower extremity [21]. The coper group was defined as individuals who have suffered an ankle sprain but did not show prolonged effects of the injury. To be included in the coper group, subjects must have met the following criteria: (1) a history of a lateral ankle sprain that required immobilization and/or non-weight bearing for at least three days, (2) no episodes of the ankle giving way and/or feeling of instability for at least 12 months prior to enrolment in this study, (3) free of cerebral concussions, vestibular disorders, and lower extremity injuries for the previous six months, and (4) no prior rehabilitation or balance training [5,21,22,23]. Inclusion criteria for the stable ankle group were the same as for the coper group, but these individuals must have never sustained an ankle injury [5]. In addition to inclusion criteria, all subjects completed the Cumberland Ankle Instability Tool (CAIT) and Foot and Ankle Ability Measure Activities of Daily Living (FAAM ADL). Subjects with stable ankles should have a score of less than 24 on the CAIT. In addition, the coper and stable ankle groups should have a score of more than 90% on the FAAM ADL subscale [5,21,23]. All participants gave written informed consent approved by the University Institution Review Board. A priori power analysis performed on similar maximum reach distance variables during SEBT determined that an average of 20 participants per group is necessary (range = 15–35 participants) [24,25].

### 2.2. Instrumentation for Kinematics

**Figure 1:**
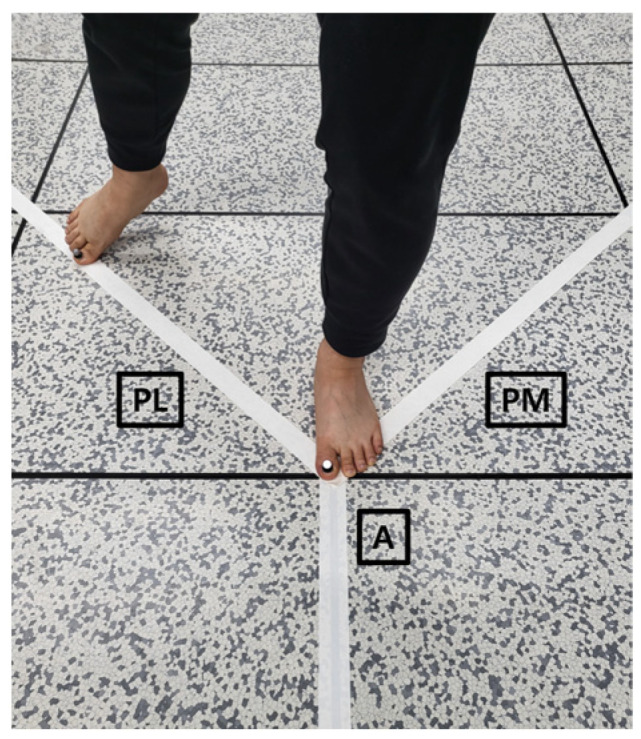
Star Excursion Balance Test (SEBT) in anterior (A), posteromedial (PM), and posterolateral (PL) directions with reflective markers on the right and left big toes.

A 3-dimensional motion-capture system (VICON MXF20, VICON Motion System, Centennial, CO, USA) and a software (Nexus version 1.8.5; VICON Motion System) were used to collect kinematic data for velocity. Reflective markers were attached to the top of the right and the left big toes to determine zero velocity in each direction (Figure 1). Each of the three zero velocities in each direction indicates the starting point just before the big toes initiated from the center of the grid, the middle point right after the big toe reached maximum distance, and the ending point right after the big toe returned back to the center of the grid, in this order. A trial of the SEBT was counted between the first (starting point) and third (ending point) zero velocities in each direction. The motion capture system was used to detect time events and reach distance during the SEBT.

### 2.3. Instrumentation for Electromyography

**Figure 2:**
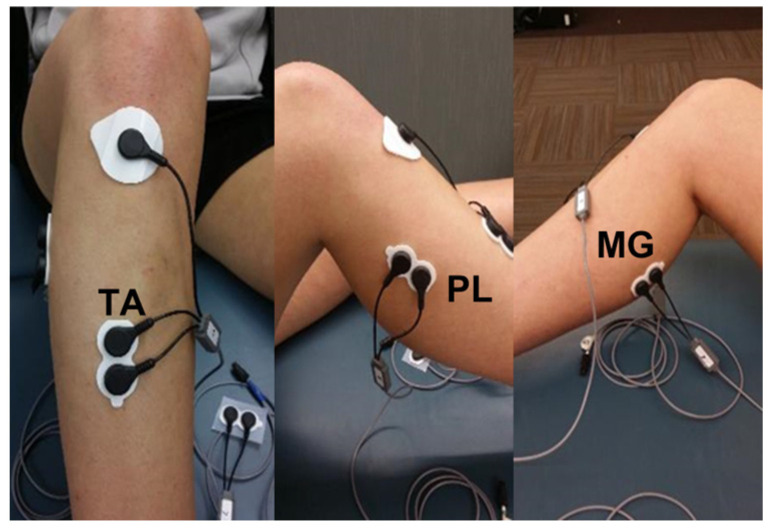
The placement of the surface electromyography electrodes for tibialis anterior (TA), fibularis longus (FL), and medial gastrocnemius (MG).

Surface EMG signals from the TA, FL, and MG were collected with the wireless surface EMG system (Telemyo 2400 G2, Noraxon, Scottsdale, AZ, USA), including a 10–500 Hz band pass filter. EMG data were sampled at 1000 Hz. All subjects’ skin was cleaned with alcohol pads prior to electrode placement. Surface electrodes were placed on the subject’s middle portion of the TA, FL, and MG belly, and a reference electrode was placed over the tibia tuberosity (Figure 2). Subjects performed one maximum voluntary isometric contraction (MVIC) for 5 s using an isokinetic dynamometer for plantarflexors (PF), evertors (EV), and dorsiflexors (DF) to normalize the mean amplitude of EMG data (% of MVIC). MVIC for the PF and DF was performed in a seated position with 90 degrees of the seatback and 90 degrees of the ankle (neutral position). Verbal encouragement was given during the performance of MVIC. A 5 min rest was given prior to the performance of the SEBT. A customized MATLAB program (The Math Works, Inc, Natick, MA, USA) was used to calculate the root mean square (RMS) of the middle 3 s of MVIC of the PF and the DF for normalization of EMG data. EMG data were only collected between the starting and ending points in each direction for each trial during the SEBT. The mean of the mean amplitudes from all three trials of the SEBT in each direction was normalized to the MVIC (% of MVIC) for all three muscles.

### 2.4. Star Excursion Balance Test

For copers and individuals with chronic ankle instability, subjects stood barefoot on their injured leg in the center of a grid. The leg tested in the stable ankle group matched the leg tested in the CAI group. The A, PM, and PL direction lines were marked at an angle of 45 degrees from the center of the grid. Subjects were instructed to reach their big toe of the non-injured side along the three lines as far as possible and then to reach their toe back to the center of the grid while maintaining a single-leg stance on their measured leg [26]. Subjects kept their hands on their waist while fully keeping their measured foot on the ground without transference of weight during the performance of the SEBT. No further instructions about technique or posture were given as long as subjects kept their hands on their hips. The rest interval between trials was 10 s. The maximal reach distances for each direction were measured. These directions are the most reliable outcome measurements to determine lower extremity impairments [19]. The test order of reach direction was randomized and counterbalanced. Maximum reach distance (MRD) was measured and normalized by the subject’s leg length. Normalization was performed by dividing the maximum reach distance by a subject’s leg length (length was measured between the anterior superior iliac spine and center of medial malleolus) and then multiplying by 100 [27]. A total of seven trials of the SEBT were completed in each of the three directions, with the first four being practice trials [28]. Trials were discarded and repeated if participants moved their hands from their hips, moved their foot from the original foot position, or failed to touch the line with the reaching foot during the trial.

### 2.5. Procedure

Subjects required one visit and rode a stationary bike for 5 min as a warm-up exercise before testing. After the warm-up, surface electrodes for EMG were placed on the subject’s middle portion of the TA, FL, and MG belly. The subject performed one MVIC for 5 s for PF, EV, and DF. Prior to the SEBT, reflective markers were placed on top of the right and left big toes of the subjects for MRD. Subjects performed 4 practice trials of the SEBT in each direction (AM, M, PM), with 10 s rest between trials before testing began. Three consecutive test trials were finally performed in each direction.

### 2.6. Statistics

The mean of three trials for maximum reach distance and the mean amplitude in each direction were calculated. Separate two-way analyses of variance (ANOVA) for normalized maximum reach distance (NMRD) and normalized mean amplitude (NMA) of the TA (NMA_TA), FL (NMA_FL), and MG (NMA_MG) were calculated to investigate a group effect (stable ankle, coper, CAI) and a direction effect (A, PM, PL). Post hoc comparisons using Fisher’s Least Significant Difference were performed to assess specific differences when there was a significant difference among population groups or direction groups. Effect sizes (partial eta-squared) of significances for the main effects were reported. The level of significance was set at 0.05 for all comparisons.

## 3. Results

No statistically significant differences (*p* > 0.05) in height, mass, and age were found between the groups. The Cumberland Ankle Instability Tool (CAIT) and Foot and Ankle Ability Measure Activities of Daily Living (FAAM ADL) scores for individuals with CAI were statistically lower than those for copers (CAIT: *p* < 0.001, FAAM ADL: *p* < 0.05) and individuals with stable ankles (CAIT: *p* < 0.001, FAAM ADL: *p* < 0.05).

### 3.1. Normalized Maximum Reach Distance (NMRD)

**Figure 3:**
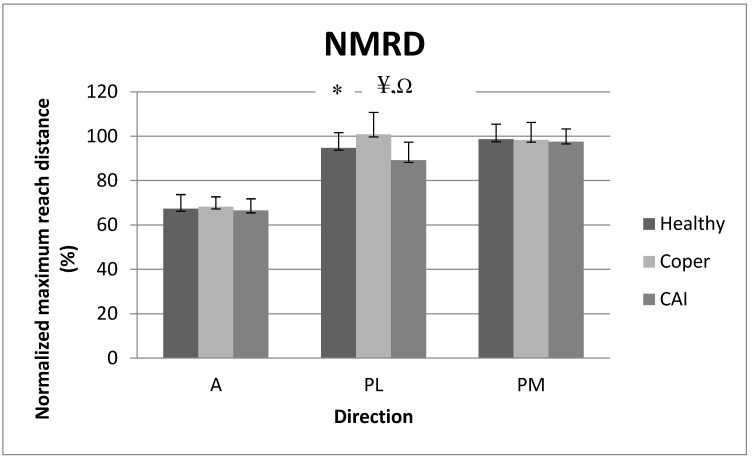
Normalized maximum reach distance (NMRD) among the three populations in the A, PL, and PM directions. A: anterior, PL: posterolateral, PM: posteromedial. ¥ *p*-value ≤ 0.05: coper > healthy in PL, Ω *p*-value ≤ 0.05: coper > CAI in PL. * *p*-value ≤ 0.05: healthy > CAI in PL.

There was a significant interaction for NMRD (F_2171_ = 3.60, *p* = 0.01), only in the PL direction. Post hoc comparisons revealed that copers had greater NMRD than both individuals with stable ankles (*p* < 0.01) and those with CAI (*p* = 0.01), and individuals with stable ankles also performed better than those with CAI (*p* = 0.01) in the PL direction. Figure 3 reports the means (SD) for NMRD.

### 3.2. Mean Amplitude of Tibialis Anterior (TA)

**Figure 4:**
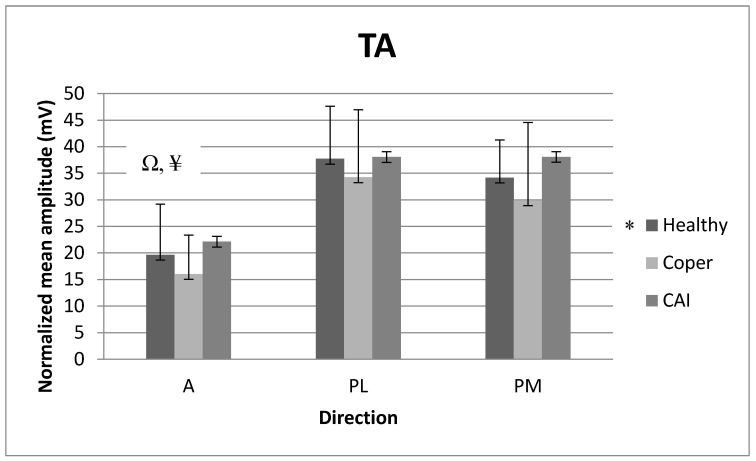
Normalized mean amplitude (NMA) for tibialis anterior (TA) among the three populations in the A, PL, and PM directions. A: anterior, PL: posterolateral, PM: posteromedial. * *p*-value ≤ 0.05: healthy > coper, Ω *p*-value ≤ 0.05: A < PL, ¥ *p*-value ≤ 0.05: A < PM.

There was no significant interaction for NMA_TA (F_2171 = 0.24,_ *p* = 0.91). However, there was a main group effect for NMA_TA (F_2171_ = 4.93, partial eta-squared = 0.06, *p* = 0.01). Post hoc comparisons revealed that individuals with CAI (*p* = 0.01) and those with stable ankles (*p* = 0.05) showed greater NMA_TA than copers regardless of the direction. There was also a main direction effect for NMA_TA (F_2171_ = 47.90, partial eta-squared = 0.08, *p* < 0.01). Post hoc comparisons revealed that the PM (*p* < 0.01) and PL (*p* < 0.01) directions showed greater NMA_TA than the A direction regardless of the population. Figure 4 reports the means (SD) for NMA_TA.

### 3.3. Mean Amplitude of Fibularis Longus (FL)

**Figure 5:**
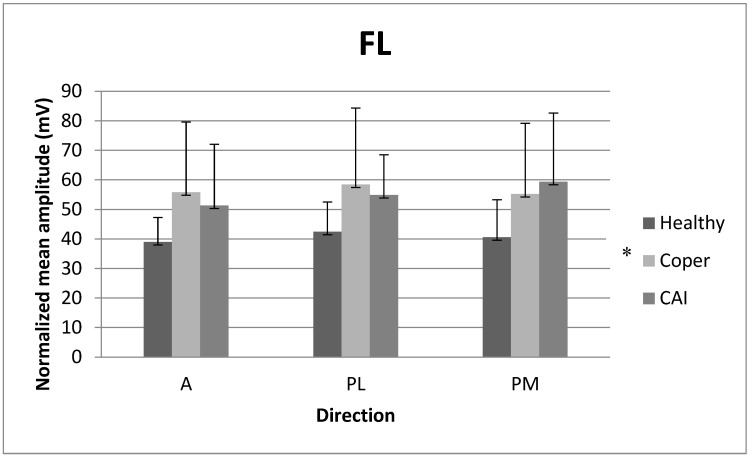
Normalized mean amplitude (NMA) for fibularis longus (FL) among the three populations in the A, PL, and PM directions. A: anterior, PL: posterolateral, PM: posteromedial. * *p*-value ≤ 0.05: coper > healthy.

There were no significant interaction (F_2171_ = 0.34, *p* = 0.85) or main direction effects (F_2171_ = 0.53, *p* = 0.58) for NMA_FL. However, there was a main group effect for NMA_FL (F_2171_ = 12.59, partial eta-squared = 0.10, *p* < 0.01). Post hoc comparisons revealed that copers (*p* < 0.01) showed a greater NMA_FL than individuals with stable ankles regardless of the direction. Figure 5 reports the means (SD) for NMA_FL.

### 3.4. Mean Amplitude of Medial Gastrocnemius (MG)

**Figure 6:**
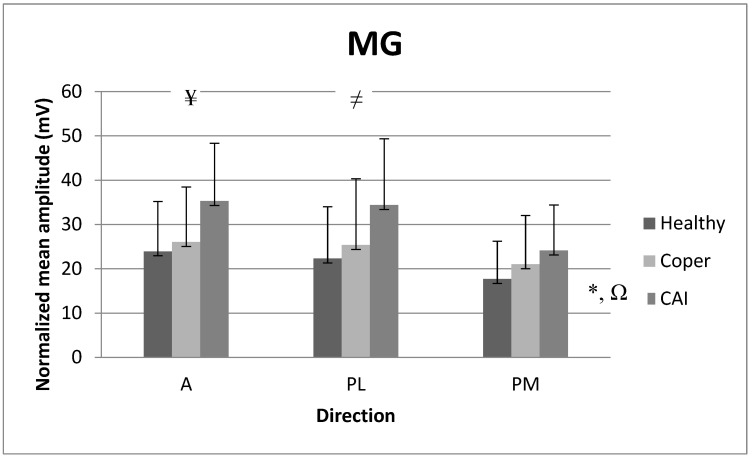
Normalized mean amplitude (NMA) for medial gastrocnemius (GA) among the three populations in the A, PL, and PM directions. A: anterior, PL: posterolateral, PM: posteromedial. * *p*-value ≤ 0.05: CAI > coper, Ω *p*-value ≤ 0.05: CAI > healthy, ¥ *p*-value ≤ 0.05: A > PM, ≠ *p*-value ≤ 0.05: PL > PM.

There was no significant interaction for NMA_MG (F_2171_ = 0.49, *p* = 0.74). However, there was a main group effect for NMA_MG (F_2171_ = 10.64, partial eta-squared = 0.12, *p* < 0.01). Post hoc comparisons revealed that individuals with CAI showed greater NMA_MG than copers (*p* < 0.01) and healthy individuals with stable ankles (*p* < 0.01) regardless of the direction. There was also a main direction effect for NMA_MG (F_2171_ = 6.64, partial eta-squared = 0.08, *p* < 0.01). Post hoc comparisons revealed that the A (*p* < 0.01) and PL (*p* < 0.01) directions showed greater NMA_MG than the PM direction regardless of the population. Figure 6 reports the means (SD) for NMA_MG.

## 4. Discussion

### 4.1. Reach Distance Analysis

The purpose of this study was to assess the impact of the three muscles on performance of the SEBT in the three directions among CAI, coper, and control groups. We hypothesized that reach distance would be greater in individuals with stable ankles than individuals with CAI and copers. In our results, individuals with CAI demonstrated a smaller reach distance than copers and individuals with stable ankles in the PL direction. The PL direction was the most distinguishable of the three directions because it requires the ankle joint to perform much more difficult tasks than the other directions [29]. Moreover, individuals with CAI have shown that they cannot fully pronate their foot in walking because they are in a more plantar-flexed position which limits the joint’s ability to pronate [30]. This may be the reason why individuals with CAI who may be in a plantar-flexed position during the performance of the SEBT demonstrated the smallest reach distance in the PL direction, as the PL direction does require the pronation of the foot in this study.

Interestingly, as our results show that copers perform better than individuals with stable ankles, this presents conflicting findings. Steib et al. reported that copers had nearly identical dynamic postural control during the SEBT to individuals with stable ankles, but individuals with CAI were still lower than both copers and individuals with stable ankles [6]. This agrees with another study by Steib et al., which found that copers are similar to individuals with stable ankles during the SEBT and after jump-landing tasks [7]. Individuals with CAI also presented decreased invertor and evertor strength when compared to copers and individuals with stable ankles, but copers and individuals with stable ankles showed no significant differences [31]. We believe that copers develop some compensatory factors that help them become more efficient than individuals with stable ankles. Bowker et al. found that copers have increased motor neuron recruitment which helps them to overcome the events that lead to developing CAI. It is unknown as to why individuals with CAI do not recruit more motor neurons to compensate for their deficits as well [32]. Additionally, there is a feed-forward motor control mechanism that acts to anticipate any deviations and to assist in protecting joints from potential damage [33]. Thus, the combination of this feed-forward mechanism and the increased motor neuron recruitment may activate the muscle more than usual during any movement, which leads us to believe that this is how copers present greater distances than individuals with stable ankles. As individuals with stable ankles never sustained an injury, they did not develop any coping mechanisms that led to increased motor neuron recruitment.

### 4.2. EMG Analysis

Mean amplitude is important to look at during the SEBT because it can assist us in interpreting which muscles are activated the most or the least to help achieve stabilization in each direction. With the decline in proprioception and muscle strength, muscles may need to activate more to compensate for the deficits. We expected muscle activation to be greater in individuals with CAI and copers than in individuals with stable ankles. Our results partially supported our hypothesis. We found that the muscle activation for the TA for individuals with CAI and those with stable ankles was indeed greater than that for copers. This result oppositely corresponded to the results of reach distance. As described before, individuals with CAI and those with stable ankles who demonstrated smaller reach distance than copers may demand increased motor unit recruitment compared to copers to complete the performance of the SEBT. This study also showed that the PL and PM directions have greater TA activation than the A direction, regardless of the population. Gabriner et al. also found that the PL and PM directions were more distinguishable compared to the A direction [9]. They stated that the PL and PM directions might require more muscle strength and postural control than the A direction [9]. Therefore, the PL and PM directions may demand greater activation of the TA compared to the A direction during the performance of the SEBT. Another plausible explanation is that the PL and PM directions, which require more plantarflexion than the A direction, could lead to eccentric contraction of the TA to maintain balance while performing the SEBT. Thus, the eccentric contraction that the posterior direction demands may result in the increased activation of the TA compared to concentric contraction, which the A direction requires.

As for the FL, our results show that individuals with CAI and copers had greater muscle activation than individuals with stable ankles, regardless of the direction. The FL is the strongest of the evertor muscles that is the main producer of eversion of the foot and has a reflex mechanism to prevent an inversion sprain [11,34]. Moreover, studies have shown that the FL muscle strength deficit is most evident and has the strongest relationship with individuals with CAI [11,35]. Brown et al. concluded that increased FL activation is necessary to compensate for the decrease in strength due to a history of ankle sprain so that individuals with CAI and copers can prevent another ankle sprain [36]. Although we agree that the FL is significant in showing the correlation in individuals with CAI, we also believe that the FL is equally important in copers because they both suffer the same mechanism of an ankle sprain. Therefore, the increased activation of the FL may be required in individuals with CAI and copers compared to individuals with stable ankles to maximally reach distance during the SEBT.

We found that the activation of the MG was greater in individuals with CAI than copers and individuals with stable ankles and in the A and PL directions compared to the PM direction. Previous studies identified that individuals with CAI demonstrated more MG activation during the toe-off stage and that the foot tends to be more plantar-flexed during a landing task compared to individuals with stable ankles [30,37,38]. According to these previous studies, we may speculate that individuals with CAI tend to produce greater MG muscle activation than individuals with stable ankles due to ankle sprain mechanism during any functional task, including the SEBT. When the center of gravity (COG) shifts backward, the anterior muscles must activate more to prevent someone from falling [39]. Oppositely, the shifts in the COG in the A direction may require greater MG activation in the posterior muscles rather than the PL or PM directions to perform the SEBT. Eltoukhy et al. demonstrated that the subject had to flex and laterally rotate the trunk while reaching the PL direction behind the stance leg to perform the SEBT [40]. The posterior direction relies more on strength and postural control than the A direction [9]. A possible explanation for greater MG activation in the PL direction is that greater MG activation may be attributed to the complex body movement required when reaching the PM direction. As we are currently the only study to measure MG activation in copers, further research is necessary to investigate soleus and lateral gastrocnemius as the posterior muscles for a better understanding during the SEBT in the PL and PM directions.

## 5. Conclusions

This study is the first to investigate how the TA, FL, and MG muscles of the ankle joint respond to the performance of the SEBT in the A, PL, and PM directions. Based on the results, copers demonstrated greater NMRD in the PL direction compared to individuals with stable ankles and those with CAI because of a compensatory mechanism that may have occurred in copers following an ankle sprain. On the other hand, the PL direction of the SEBT could be a more sensitive and reliable direction to distinguish neuromuscular functions among these groups. Since our results showed that individuals with CAI demonstrated greater MG activation than copers and that the PL direction required greater activation of MG, individuals with CAI may need greater MG activation to increase control of ankle instability during the SEBT in the PL direction. Thus, clinicians should consider how to rehabilitate their patients with a history of at least one ankle sprain to improve postural stability during balance tasks [41]. This phenomenon may help to understand why individuals with an ankle sprain did not develop CAI. This study only investigated muscle activation as a potential contributor at the ankle. The addition of range of motion and skeletomuscular electrical activity at the ankle, knee, hip, and trunk complex was considered to contribute to CAI [42]. Therefore, future studies need to investigate these factors to allow for a better understanding of our results in copers compared to individuals with stable ankles and CAI for each reach direction. The small sample size was considered a limitation of this study. This study is limited because the mean of EMG data was measured throughout the entire reach. Since the surface of EMG measurement can depend on muscle length, it would be better for our understanding of muscle activation if the reach was broken down into the reach and return phase for the mean EMG data.

## Data Availability

Data that support the findings of this study are available from the corresponding author upon reasonable request.
